# Molecular phylogenetic study of the tribe Tropidieae (Orchidaceae, Epidendroideae) with taxonomic and evolutionary implications

**DOI:** 10.3897/phytokeys.140.46842

**Published:** 2020-02-19

**Authors:** Izai A.B. Sabino Kikuchi, Paul J.A. Keßler, André Schuiteman, Jin Murata, Tetsuo Ohi-Toma, Tomohisa Yukawa, Hirokazu Tsukaya

**Affiliations:** 1 Universiteit Leiden, Hortus botanicus Leiden, PO Box 9500, Leiden, 2300 RA, The Netherlands; 2 Science Directorate, Royal Botanic Gardens, Kew, Richmond, Surrey, TW9 3AB, UK; 3 Botanical Gardens, Graduate School of Science, The University of Tokyo, 3-7-1 Hakusan, Bunkyo-ku, Tokyo, 112-0001, Japan; 4 Tsukuba Botanical Garden, National Science Museum, 4-1-1 Amakubo, Tsukuba, 305-0005, Japan; 5 Department of Biological Sciences, Faculty of Science, The University of Tokyo, 7-3-1 Hongo, Bunkyo-ku, Tokyo, 113-0033, Japan; 6 Bio-Next Project, Okazaki Institute for Integrative Bioscience, National Institutes of Natural Sciences, Yamate Build. #3, 5-1, Higashiyama, Myodaiji, Okazaki, Aichi, 444-8787, Japan

**Keywords:** *
Corymborkis
*, *
Kalimantanorchis
*, mycoheterotrophy, phylogeny, *
Tropidia
*

## Abstract

The orchid tribe Tropidieae comprises three genera, *Tropidia*, *Corymborkis* and *Kalimantanorchis*. There are three fully mycoheterotrophic species within Tropidieae: *Tropidia saprophytica*, *T. connata* and *Kalimantanorchis nagamasui*. A previous phylogenetic study of *K. nagamasui*, based only on plastid *matK* data, placed *K. nagamasui* outside the clade of *Tropidia* and *Corymborkis* without support. In this study, we performed phylogenetic analyses using a nuclear ribosomal DNA spacer (ITS1-5.8S-ITS2), a low-copy nuclear coding gene (*Xdh*) and a mitochondrial intron (*nad1b-c* intron) to study the phylogenetic relationships within Tropidieae. We included six photosynthetic and all three fully mycoheterotrophic Tropidieae species. The resulting phylogenetic trees placed these fully mycoheterotrophic species inside the *Tropidia* clade with high support. In our trees, these three species do not form a monophyletic group together, because the photosynthetic *T. graminea* is nested amongst them. Our results also suggest that the loss of photosynthetic ability occurred at least twice in *Tropidia*.

## Introduction

The tribe Tropidieae is one of the earliest diverging clades in subfamily Epidendroideae (Orchidaceae) and currently contains three genera: *Corymborkis* Thouars, *Tropidia* Lindl. and *Kalimantanorchis* Tsukaya, M.Nakaj. & H.Okada (Tsukaya et al. 2011; Freudenstein and Chase 2015; Koch et al. 2016). *Corymborkis* comprises eight species and has a pantropical distribution (Rasmussen 1977; Govaerts et al. 2019). About 30 species are recognised in *Tropidia* and most of the species occur in tropical Asia and Australasia; the Neotropical *T. polystachya* (Sw.) Ames is the only species outside Asia and Australasia (Jones and Bolger 1988; Kumar et al. 2015; Koch et al. 2016; Ormerod 2018). *Kalimantanorchis* is the most recently established genus in the tribe with the single fully mycoheterotrophic species *K. nagamasui* Tsukaya, M.Nakaj. & H.Okada from Sabah and West Kalimantan in Borneo (Tsukaya et al. 2011; Suetsugu et al. 2017a). Two other fully mycoheterotrophic species are reported in Tropidieae; both belong to *Tropidia*: *T. saprophytica* J.J.Sm. and *T. connata* J.J.Wood & A.L.Lamb. *Tropidia saprophytica* has been recorded from Sabah and Sarawak and *T. connata* from Sabah and West Kalimantan (Wood 1988; Kikuchi and Tsukaya 2017). Wood (1984) established the fully mycoheterotrophic genus *Muluorchis* J.J.Wood together with the description of *Muluorchis ramosa* J.J.Wood. He later found that the species is conspecific with *T. saprophytica* and synonymised *Muluorchis* with *Tropidia* (Wood 1988). Wood and Lamb later described another fully mycoheterotrophic species, *T. connata* (Wood and Cribb 1994). *Tropidia connata* is easily distinguished from *T. saprophytica* by the zigzag inflorescence, connate lateral sepals and shortly spurred lip (Wood and Cribb 1994). More recently, *Kalimantanorchis* was established as a new genus, based on its morphological characters and molecular phylogenetic data (Tsukaya et al. 2011). The subterranean tuberous structure of *Kalimantanorchis* had never been observed in the other two fully mycoheterotrophic *Tropidia* species, although we now know that a tuberous structure occurs at least in *T. connata* as well (Tsukaya et al. 2011; Kikuchi and Tsukaya 2017). Kikuchi and Tsukaya (2017) suggested that the tuberous structures of the fully mycoheterotrophic Tropidieae species may be equivalent to the tuber-like nodules of other leafy *Tropidia* (Yeh et al. 2009, Chun-Kuei et al. 2013). On the other hand, Tsukaya et al. (2011) provided a molecular phylogenetic tree based only on plastid *matK* data, in which *Kalimantanorchis* was placed outside the clade comprising *Tropidia* and *Corymborkis*.

The study of mycoheterotrophy is hampered by two major obstacles (Merckx et al. 2013a). One is the rarity of many fully mycoheterotrophic species: several have been found only once or twice in the field. Due to the scarcity of adequate material, for some of the species, information is lacking even at the basic level of morphology and anatomy. The other major obstacle is the elevated plastid DNA substitution rates usually occurring in fully mycoheterotrophic plants. In these plants, the leaves are normally reduced to scales or sheaths and they are achlorophyllous (Tsukaya 2018). Most of the plastid genes of fully mycoheterotrophic species are, supposedly, not functioning anymore as the plants have lost the ability to conduct photosynthesis and often experienced higher mutation rates and deletions in these genes (Freudenstein and Senyo 2008; Delannoy et al. 2011). Although some fully mycoheterotrophic species still retain amplifiable plastid DNA regions, they can be difficult to align and analyse due to these elevated substitution rates (Merckx et al. 2013a). Since most of the phylogenies of the photosynthetic, presumably autotrophic or partially mycoheterotrophic relatives are at least partly based on plastid regions, it is sometimes impossible to relate the fully mycoheterotrophic species properly to other lineages. Therefore, the phylogenetic placement of fully mycoheterotrophic plants has often been difficult. These problems also apply to the fully mycoheterotrophic Tropidieae species.

**Figure 1.**
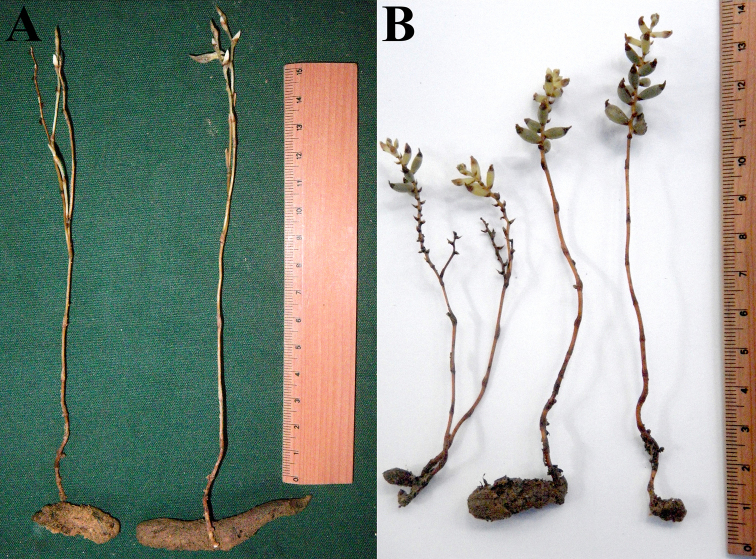
*Tropidia connata* J.J.Wood & A.L.Lamb and *Kalimantanorchis nagamasui* Tsukaya, M.Nakaj. & H.Okada. **A** Gross morphology of *T. connata* individual (specimen number 1040, collected in January, 2011 by H. Tsukaya, H. Okada and A. Soejima). **B** Gross morphology of fruiting *K. nagamasui* individual (specimen number HT1035, collected in January, 2011 by H. Tsukaya, H. Okada and A. Soejima). Scale in cm.

In 2011 and 2012, multiple specimens of *Tropidia connata* were collected by the last author (HT) in Borneo. These specimens included underground tuberous structures, which were not described in the original description (Wood and Cribb 1994) due to the incompleteness of the examined material. The examination of tuberous structures in *T. connata* clearly indicates that the tuber is a shared character between *Kalimantanorchis nagamasui* and *T. connata*, suggesting that *Kalimantanorchis* may not be distinct from *Tropidia* (Kikuchi and Tsukaya 2017) (Fig. 1). As already mentioned, Tsukaya et al. (2011) provided a phylogenetic analysis based on a plastid region (*matK*), which placed *Kalimantanorchis* outside the *Tropidia* + *Corymborkis* clade. However, this result had low support, possibly because of a highly elevated substitution rate of *matK* in *Kalimantanorchis*.

Based only on the morphological findings of Kikuchi and Tsukaya (2017), Ormerod and Juswara (2019) synonymised *Kalimantanorchis* with *Tropidia*; however, they did not provide molecular evidence to counter the findings of Tsukaya et al. (2011). In this study, we were able to include all three fully mycoheterotrophic species within a broader sampling of Tropidieae compared to the previous phylogenetic analysis of Tsukaya et al. (2011) and conducted molecular phylogenetic analyses using a nuclear ribosomal DNA spacer (ITS1-5.8S-ITS2), a low-copy nuclear coding gene (*Xdh*) and a mitochondrial intron (*nad1b-c* intron) to clarify the placement of the fully mycoheterotrophic species.

## Methods

### Taxon sampling and plant material

Nine species of Tropidieae were included in this study (six species of *Tropidia*, one of *Kalimantanorchis* and two of *Corymborkis*). We sampled all three fully mycoheterotrophic species. Apart from *Tropidia polystachya* occurring in the Neotropics, all sampled *Tropidia* species are native to Asia. We selected *Sobralia rosea* Poepp. & Endl. (Epidendroideae: Sobralieae) as an outgroup taxon for the phylogenetic analyses considering that Sobralieae is sister to the rest of Epidendroideae, except for Neottieae (Freudenstein and Chase 2015; Givnish et al. 2015). We obtained three sequences of *Sobralia rosea* from GenBank. We used five DNA samples from the DNA Bank of the Royal Botanic Gardens, Kew. Four samples were collected in the field and extracted in this study. We provided the sample and voucher information in Table 1.

**Table 1.** Voucher information and GenBank accessions of samples used in phylogenetic analyses. Herbarium codes follow Thiers (2019). Samples with NA were extracted in this study, except for *S. rosea*. Sequences of *S. rosea* were obtained from GenBank.

**Taxon**	**Voucher information**	**Kew DNA Bank ID**	**ITS**	**Xdh**	**nad1 b-c intron**
*Corymborkis corymbis* Thouars	10041 (REU)	23681	MH596711	MH594866	MH594874
*Corymborkis veratrifolia* (Reinw.) Blume	Tsukaya et al., B200608289 (BO)	NA	MH596710	MH594865	MH594875
*Kalimantanorchis nagamasui* Tsukaya, M.Nakaj. & H.Okada	Tsukaya et al., T31 (BO, TI)	NA	MH596709	MH594864	MH594873
*Sobralia rosea* Poepp. & Endl.	Romano, Sob5 and Szlachetko	NA	KT923827	KT923862	EF464132
*Tropidia bambusifolia* (Thwaites) Trimen	without number	17721	MH572221	MH594863	MH594872
*Tropidia connata* J.J.Wood & A.L.Lamb	Tsukaya et al., 224 (TI)	NA	MH596708	MH594862	MH594871
*Tropidia graminea* Blume	Duangjai, 40 (BRUN, K)	21775	MH596707	MH594861	MH594870
Tropidia nipponica var. hachijoensis F.Maek. & Yokota	Matsumoto, 466 (TNS)	NA	MH596706	MH594860	MH594869
*Tropidia polystachya* (Sw.) Ames	89001 (MWC)	O-211	MH596705	MH594859	MH594868
*Tropidia saprophytica* J.J.Sm.	Cameron	O-798	MH596704	MH594858	MH594867

### DNA extraction, PCR amplification and sequencing

DNA extraction was conducted for the four samples obtained in the field using FTA cards (Whatman, Tokyo, Japan) and DNeasy kit (Qiagen, Hilden, Germany). Amplification for the nuclear ITS, *Xdh* and mitochondrial *nad1b-c* intron, was performed for nine samples using TaKaRa ExTaq polymerase (TaKaRa Bio, Shiga, Japan) and DreamTaq DNA Polymerases (Thermo Fisher, Epsom, UK). The following primer sets were used: ITS1 and ITS4 for ITS1-5.8S-ITS2 (White et al. 1990), X502 and X1599R for *Xdh* (Górniak et al. 2010) and nad1 exon B and nad1 exon C for *nad1 b-c* intron (Demesure et al. 1995). For ITS, the thermal cycling protocol began with 3 min initial denaturation at 94°C, followed by 35 amplification cycles, each with 15 s at 94°C, 30 s at 44°C and 40 s at 72°C, which was concluded by a final extension at 72°C for 7 min. For *Xdh*, the thermal cycles were as follows: initial denaturation at 94°C for 2 min, 6 touchdown cycles of 45 s at 94°C, 45 s at 53°C and 90 s at 72°C, reducing one degree per cycle, which was followed by 28 cycles of 45 s at 94°C, 45 s at 47°C and 90 s at 72°C and a final extension at 72°C for 5 min. For *nad1 b-c* intron, the thermal cycles were set as follows: pre-melting for 3 min at 94°C, followed by 35 cycles of 15 s at 94°C, 30 s at 50°C and 2 min at 72°C and a final extension at 72°C for 7 min. Cycle sequencing was conducted using Big Dye Terminator v3.1 Cycle Sequencing Kit (Applied Biosystems, Foster City, CA, USA) with the same primers used for PCR reactions. We additionally used nad1M as an internal primer for sequencing *nad1 b-c* intron (see Freudenstein and Chase 2001). Newly generated 27 sequences were deposited in GenBank and their accession numbers are listed in Table 1 with the accession numbers of the three sequences of *Sobralia*, which were obtained from GenBank.

### Phylogenetic analyses

In total, 30 sequences were edited and assembled using Geneious Prime 2019.2.1 (https://www.geneious.com) (Kearse et al. 2012). Sequence alignments for each region were generated with the MUSCLE alignment tool (Edgar 2004), installed in Geneious Pro using the default setting. Phylogenetic relationships were inferred for the concatenated alignments of the three markers (ITS, *nad1b-c* intron, *Xdh*). The best partitioning scheme was selected by the Bayesian Information Criterion (BIC) using PartitionFinder 2.1.1 (Guindon et al. 2010; Lanfear et al. 2016). The best partitioning and substitution models for each partition were estimated as follows: ITS (K80+G), *nad1 b*-c intron (K81UF), *Xdh* first and second codon (K80), *Xdh* third codon (HKY). The Maximum Likelihood (ML) analysis was conducted using IQ-TREE 1.6.12 (Nguyen et al. 2015). We obtained branch supports by the ultrafast bootstrap with 1000 replicates (Hoang et al. 2018). The Bayesian Inference (BI) analysis was performed using MrBayes 3.2.7a (Ronquist et al. 2012). The BI analysis was run for 50000 generations. Trees were sampled every 100 generations of the MCMC chain. By default, MrBayes discarded the first 25% samples from the cold chain. The average standard deviation of split was checked at the end of the run. Using the sump command, the model parameters were also checked for the convergence diagnostic, the Potential Scale Reduction Factor (PSRF). All the PSRF values were close to 1.0. A 50% majority rule consensus tree was estimated using the sumt command to obtain the posterior probabilities for each clade. Generated trees were visualised using FigTree 1.4.3 (Rambaut 2017). Clades with over 85% bootstrap (BS) value or 0.95 Bayesian Posterior Probability (PP) were considered strongly supported. Clades with over 75% BS or 0.90 PP support were considered moderately supported. Clades with lower support values were not regarded as reliably supported clades.

## Results

The aligned sequence lengths for each region were 669 bp (ITS), 1389 bp (*nad1b-c* intron) and 843 bp (*Xdh*). We separately conducted ML analyses for the three regions and none of the incongruences in topology was significantly supported between the trees. Therefore, the combined usage of the three markers was justified.

**Figure 2.**
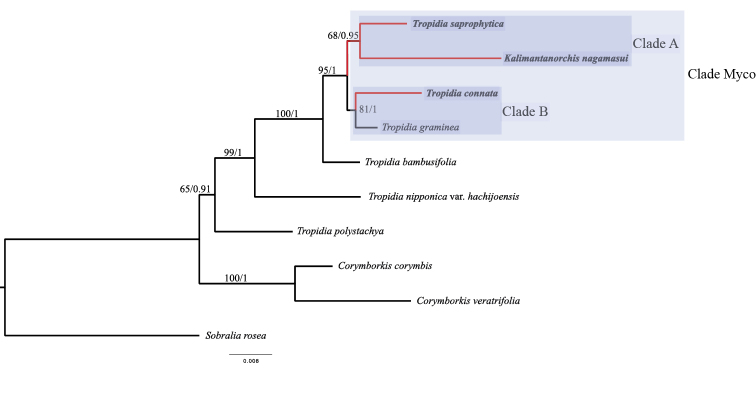
Phylogenetic relationships of Tropidieae based on Maximum Likelihood analysis on three non-plastid regions (ITS, *nad1 b-c* intron and *Xdh*). Numbers at nodes are Bootstrap Percentages obtained by Maximum Likelihood analysis and Bayesian Posterior Probabilities, respectively. Fully mycoheterotrophic species are shown in bold. Red branches indicate mycoheterotrophic origins. The scale bar below the tree indicates the substitution rate.

The positions of all the taxa were consistent in both trees resulting from the ML and BI analyses for the combined alignments. As the topology was consistent with strong support, we added the bootstrap values obtained from the BI analysis to the ML tree, which is shown in Figure 2.

In both the ML and BI trees, *Corymborkis* species form a monophyletic clade (BS = 100%, PP = 1). *Tropidia* species also form a monophyletic clade with the Neotropical *T. polystachya* as the first diverging species with a moderately high PP support (0.91) but with a low BS support (65%) (Fig. 2). In our trees, *T. polystachya* is sister to all Asian *Tropidia* species. In this Asian *Tropidia* clade, the East Asian Tropidia nipponica var. hachijoensis F.Maek. & Yokota is the first diverging species, followed by the Sri Lankan *Tropidia bambusifolia* (Thwaites) Trimen. The three fully mycoheterotrophic Tropidieae species form a clade (clade Myco, hereafter) with the photosynthetic *Tropidia graminea* Blume. In clade Myco, the clade which includes *K. nagamasui* and *T. saprophytica* (clade A, hereafter) is sister to the clade formed by *T. connata* and *T. graminea* (clade B, hereafter) with high support (BS = 95%, PP = 1).

## Discussion

### Monophyly of Corymborkis and Tropidia

*Corymborkis* and *Tropidia* were each inferred as monophyletic in our study. In the comparative morphological study of *Corymborkis* by Rasmussen (1977), *Tropidia polystachya* was mentioned as an exceptional *Tropidia* species because of the type of inflorescence feature, which is shared with *Corymborkis*: branched inflorescence when fully developed. As *T. polystachya* is placed as the first diverging species of *Tropidia* in our trees, we suggest that the branched inflorescence may be a symplesiomorphic character in *Corymborkis* and *T. polystachya*. As the morphology of these orchids is still little studied, it would be desirable to analyse character evolution in more detail using a broader sampling of Tropidieae.

### Evolution of non-photosynthetic feature

The evolutionary pathway to full mycoheterotrophy is hypothesised to be irreversible (Barrett et al. 2014). Once the ability to perform photosynthesis is lost, the photosynthetic genes are not expected to be regained (Lam 2016). In our results, *Tropidia graminea*, a photosynthetic species with well-developed leaves, is placed as a sister taxon to fully mycoheterotrophic *T. connata* in clade B, which is sister to clade A. Granted that the evolution of the non-photosynthetic feature is irreversible, we hypothesise that the ancestral character of clade A and clade B is photosynthetic. Therefore, this result indicates that non-photosynthetic features independently evolved at least twice from photosynthetic ancestors in the *Tropidia* clade (Fig. 2). However, the branch lengths of the basal nodes of clade A and clade B are short and it might be the case that long branch attraction or the incongruences amongst datasets influenced this topology. We can only test this if we increase the number of sampled species from Asian *Tropidia* and include more markers to conduct phylogenetic analyses.

Flowering plant genera with multiple transitions to full mycoheterotrophy have rarely been reported to date. *Hexalectris* Raf. (Orchidaceae) and *Burmannia* L. (Burmanniaceae) are two of the few genera known to have multiple transitions to full mycoheterotrophy (Merckx et al. 2008; Barrett et al. 2019).

In Orchidaceae, more than 230 species in 43 genera are assumed to be non-photosynthetic and thus fully mycoheterotrophic (Merckx et al. 2013b). It is estimated that within Orchidaceae, full mycoheterotrophy has evolved about 30 times independently, possibly from partially mycoheterotrophic ancestors (Freudenstein and Barrett 2010). Partially mycoheterotrophic species still retain the ability to photosynthesise at the same time gaining carbon also from the mycorrhizal fungal partners and this nutritional mode of life has been suggested to be more widespread in Orchidaceae than previously assumed (Gebauer et al. 2016). Partial mycoheterotrophy has been hypothesised to be an intermediate evolutionary step from initial mycoheterotrophy towards full mycoheterotrophy in Orchidaceae (Merckx et al. 2013a). For instance, it is likely that in *Cymbidium* Sw. fully mycoheterotrophic species evolved from partial mycoheterotrophic ancestors (Motomura et al. 2010). Partial mycoheterotrophy has been confirmed for many photosynthetic orchid species, mainly in subfamily Epidendroideae through isotopic analyses (Julou et al. 2005; Cameron et al. 2009; Suetsugu et al. 2017b).

So far, the occurrence of partial mycoheterotrophy in *Tropidia* has not been examined. However, all species of *Tropidia* usually grow in deep shade in the forest understorey, which supports the hypothesis that they might be partially mycoheterotrophic (Rasmussen 2005). In order to elucidate the evolutionary pathway from photosynthetic plants to fully mycoheterotrophic organisms in *Tropidia*, isotopic analyses on photosynthetic *Tropidia* species would be indispensable.

### Kalimantanorchis, a synonym of Tropidia

*Kalimantanorchis* is nested in the Asian *Tropidia* clade with high support in our study. This suggests that the previous molecular phylogenetic analysis, based only on plastid *matK* (Tsukaya et al. 2011), failed to show the phylogenetic relationships. The *matK* sequence of *Kalimantanorchis* used in that study was extremely short (501 bp) and we suspect that it also had a particularly high substitution rate, which possibly made the alignment problematic and thus caused the unsupported placement of *Kalimantanorchis* outside the *Tropidia* + *Corymborkis* clade. According to our results and the finding of the tuberous underground structures being a shared character with *T. connata* (Kikuchi and Tsukaya 2017), we cannot support the recognition of *Kalimantanorchis* as a distinct genus.

## Conclusions

According to morphological data by Kikuchi and Tsukaya (2017) and the highly supported positions of *Kalimantanorchis* inside *Tropidia* in our phylogenetic trees, we reject both grounds (i.e. the unique subterranean tuberous structure of *Kalimantanorchis* and the result of the molecular phylogenetic analysis based on *matK*) for the establishment *Kalimantanorchis* as a distinct genus suggested by Tsukaya et al. (2011). We support the synonymisation of *Kalimantanorchis* with *Tropidia*, as proposed by Ormerod and Juswara (2019).

Our results suggest that the loss of photosynthetic ability occurred at least twice in *Tropidia*. Genera with multiple transitions to non-photosynthetic full mycoheterotrophy have rarely been reported. These genera may play an important role in future studies of the evolution of mycoheterotrophy, as they may provide insights into the drivers of such transitions.
